# Protective Effect of Fasudil on Hydrogen Peroxide-Induced Oxidative Stress Injury of H9C2 Cardiomyocytes

**DOI:** 10.1155/2021/8177705

**Published:** 2021-12-01

**Authors:** Yu Zhang, Shanxin Liu, Xiaochun Li, Jian Ye

**Affiliations:** ^1^Department of Cardiology, The Second Affiliated Hospital of Zhejiang University, School of Medicine, Hangzhou, China; ^2^Department of Cardiology, The Affiliated Hospital of Hangzhou Normal University, Hangzhou, China; ^3^Department of Infectious Diseases, The Second Affiliated Hospital of Zhejiang University, School of Medicine, Hangzhou, China

## Abstract

**Objective:**

Oxidative damage is a pathological factor that causes cardiovascular damage in the clinic and is increasingly serious. This study focused on the effect of fasudil on H_2_O_2_-induced oxidative damage in cardiomyocytes.

**Materials and Methods:**

H9C2 cardiomyocytes were cultured in vitro and divided into three groups: control group (Con group), H_2_O_2_ treatment (H_2_O_2_ group), and fasudil and H_2_O_2_ cotreatment (H_2_O_2_+fasudil group). The content levels of LDH and MDA in the supernatant were detected, and the morphology of H9C2 cardiomyocytes was observed by light microscopy. 8-OHdG staining was observed by a fluorescence inversion microscope. Cell Counting Kit (CCK-8), western blotting, real-time polymerase chain reaction (RT-PCR), and enzyme-linked immunosorbent assay (ELISA) were used to investigate the effect of fasudil on the Rho/ROCK signaling pathway.

**Results:**

Our results showed that after H_2_O_2_ treatment, the H9C2 cardiomyocytes were irregular in shape and elliptical. But the morphology of the H_2_O_2_+fasudil group was similar to that of the Con group. The green fluorescence of the H_2_O_2_ group was significantly enhancer than that of the Con group, while the green fluorescence of the H_2_O_2_+fasudil group was weaker than those of the H_2_O_2_ group. By detecting the supernatant, it was found that the contents of LDH were significantly increased, and the contents of SOD and CAT in the H_2_O_2_ group were significantly decreased. And the expression of antioxidant indicators in the H_2_O_2_ group was significantly decreased by western blotting. The results of RT-PCR showed that SOD1 and SOD2 mRNA in the H_2_O_2_ group was significantly reduced, and the contents of GPX1 and GPX3 in the H_2_O_2_ group were significantly decreased by enzyme-linked immunosorbent assay (ELISA). The expression of ROCK1, ROCK2, and downstream phosphorylation of myosin phosphatase target subunit-1 (p-MYPT-1) was significantly increased in the H_2_O_2_ group, while fasudil inhibited the increase of ROCK1, ROCK2, and p-MYPT-1.

**Conclusions:**

Fasudil can inhibit the Rho/ROCK signaling pathway induced by H_2_O_2_ and reduce oxidative stress response, inhibit apoptosis, and improve antioxidant enzyme activity in H9C2 cardiomyocytes thereby delaying cell senescence.

## 1. Introduction

The rising trend of cardiovascular disease leading to human death is one of the main causes of sudden death in humans [1]. As people's quality of life improves, the incidence of many underlying diseases increases year by year, including diabetes, high blood pressure, and myocardial ischemia [2]. Among them, the incidence and mortality of cardiovascular diseases have remained at the leading level [3]. In recent years, more studies have shown that oxidative stress- (OS-) induced cardiomyocyte apoptosis plays an important role in the development of cardiovascular diseases [4]. Cardiomyocytes are a type of highly differentiated cells. Apoptosis of cardiomyocytes leads to cardiac dysfunction, which ultimately leads to irreversible changes in the heart [5]. Therefore, the search for new drugs to inhibit OS and reduce myocardial cell apoptosis is currently a hot spot in the treatment of cardiovascular diseases.

Fasudil is a novel Rho kinase inhibitor that can inhibit OS and inflammatory responses [6]. It has been reported that fasudil can directly bind to Rho-associated protein kinase (ROCK) and inhibit Rho enzyme activity, thereby attenuating ROS-induced abnormal activation of the Rho/ROCK signaling pathway [7]. Previous studies have found that fasudil can effectively reduce the expression of inflammatory factors in noninfarcted cardiomyocytes of rats with myocardial infarction [8]. Researches have reported that fasudil can regulate macrophage polarization and improve myocardial fibrosis in mice [9]. However, there are few researches on oxidative damage of cardiomyocytes induced by H_2_O_2_ in fasudil. Therefore, this research mainly discusses the effect of fasudil on H_2_O_2_-induced myocardial injury.

Rho protein is a small molecule guanylate binding protein, and Rho protein has GTPase activity and is expressed in mammalian tissue cells [10]. There are two states of Rho protein, one is the inactivation state bound to GDP (GDP-Rho), and the other is the activation state bound to GTP (GTP-Rho). ROCK is divided into two types: ROCK1 and ROCK2 [11]; activated ROCK can inhibit the activity of its downstream MYPT-1 through phosphorylation, while phosphorylated MYPT-1 can affect the contraction of blood vessels [12]. Studies have reported that the Rho/ROCK pathway plays an important role in cell movement, proliferation, and activation of cytokines [13]. Researches have confirmed that the Rho/ROCK signaling pathway is associated with cellular inflammation, OS, and apoptosis [14]. Therefore, fasudil has been studied as a specific inhibitor of the Rho/ROCK pathway in many fields.

## 2. Material and Methods

### 2.1. Cell Culture and Treatment

H9C2 cardiomyocytes (Cell Culture Center, Shanghai, China) were cultured in Dulbecco's modified Eagle's medium (DMEM) (Life Technology, Wuhan, China), medium containing 10% fetal bovine serum (FBS) (Life Technology, Wuhan, China) and 1% penicillin/streptomycin (Life Technology, Wuhan, China). H9C2 cardiomyocytes were evenly divided into three groups. When the cells were at the appropriate density, the H_2_O_2_ group and the fasudil group were treated with 200 *μ*mol/L H_2_O_2_, and the Con group was added with the same amount of DMEM for 24 h. The H_2_O_2_+fasudil group was treated with fasudil (Qianmo Biotechnology, Hubei, China) for 3 hours before H_2_O_2_ treatment.

### 2.2. Drug Preparation

Fasudil was formulated into a stock solution with physiological saline and stored in a refrigerator at -20°C. And DMEM was diluted before use.

### 2.3. Cell Counting Kit (CCK-8) Assay

The optimal concentration and time of fasudil were determined by the CCK-8 (CCK-8, Construction, Nanjing, China) method. H9C2 cardiomyocytes growing in logarithmic phase were inoculated into a 96-well plate and cultured at a density of 100 *μ*L/well for 24 h. Different concentrations of fasudil working solution were added to the plate. Each group was incubated with 10 *μ*L of CCK-8 working solution for 1 h at 1 h, 3 h, 6 h, and 12 h, and the absorbance of the three groups was measured at 450 nm with a microplate reader.

### 2.4. Lactate Dehydrogenase (LDH), Malondialdehyde (MDA), and Catalase (CAT) Levels Were Determined

The supernatants of each group of cells were collected, and the supernatant was treated with a commercial kit according to the manufacturer's instructions (Jiancheng, Nanjing, China), and the levels of LDH, MDA, and CAT were measured with a microplate reader.

### 2.5. Superoxide Dismutase (SOD) Detection

H9C2 cardiomyocytes were transferred to a 6-well plate; after treatment, the supernatant was collected and centrifuged. The SOD level in the cells was measured according to the SOD Assay Kit manual (Jiancheng, Nanjing, China).

### 2.6. RNA Isolation and Real-Time Polymerase Chain Reaction (RT-PCR)

0.5 mL of Trizol (Thermo Fisher Scientific, Shanghai, China) was added into a 24-well plate per well and shaken on ice for 10 minutes, then the liquid in each hole was collected in the EP tube without enzyme, and 0.1 mL chloroform was added to each tube, turned it upside down for 15 seconds, and placed it on top of the ice for 10 minutes. The mixture was centrifuged at 4°C (13,000 rpm, 15 min), the upper aqueous phase was aspirated, and an equal amount of isopropanol was added. The mixture was shaken for 30 seconds and allowed to stand at room temperature for 10 minutes. Then, centrifuged for another 10 minutes and discarded the supernatant. After washing the RNA pellet with 75% ethanol, it was centrifuged at 4°C (13,000 rpm, 10 min). The liquid was discarded and dissolved by the 20 *μ*L of ribonuclease-free water. RNA concentration was measured immediately, and the absorbance at 260 nm and 280 nm was measured. If the A260/A280 was between 1.8 and 2.0, the RNA quality was considered to be standard and can be used in subsequent experiments.

mRNA quantitative analysis was achieved using the Prism 7300 Sequence Detection System, using a designed reaction system, including SYBR green, positive and negative strand primers, enzyme-free water (Thermo Fisher Scientific, Shanghai, China), and template DNA. Quantitative amplification was performed under specific PCR conditions. Data were normalized using endogenous glyceraldehyde 3-phosphate dehydrogenase (GAPDH). The comparison threshold period (Ct) method, that is, the 2^-*ΔΔ*Ct^ method was used to calculate the folding magnification, and the data was analyzed by the SDS software. RT-PCR primers are shown in Table 1.

### 2.7. Western Blotting

After adding 200 *μ*L of the lysate to the 6-well plate, it was allowed to stand for 20 minutes on ice, and the liquid was collected and centrifuged (13,000 rpm, 15 min); then, the supernatant was collected. The concentration of the protein was determined by the bicinchoninic acid (BCA) (Camilo Biological, Nanjing, China) method and quantified. The protein was separated using a 10% sodium dodecyl sulfate-polyacrylamide gel, then transferred to a polyvinylidene fluoride (PVDF) membrane (Millipore, Billerica, MA, USA) for 2 hours at 4°C. 5% skim milk was prepared with Tris-buffered saline with Tween-20 (TBST) to block the specific antigen for 2 hours. After washing with TBST for 1 minute, PVDF membranes were incubated with a specific primary antibody (SOD1, Abcam, Rabbit; 1 : 3000; SOD2, Abcam, Rabbit, 1 : 3000; GPX1, US 1 : 1000, CST; GPX3, US 1 : 1000, CST; Bcl-2, Abcam, Rabbit, 1 : 2000; Bax, Abcam, Rabbit, 1 : 500; Sirt1, Bioworld, mouse, 1 : 500, China; P53, Abcam, Rabbit, 1 : 2000; ROCK1, Abcam, Rabbit, 1 : 500; ROCK2, Abcam, Rabbit, 1 : 1000; MYPT-1, Abcam, Rabbit, 1 : 1000; GAPDH US 1 : 1000 CST) at 4°C overnight. The next day, TBST was washed for 30 minutes. Specific proteins were detected by secondary antibodies and observed by the electrochemiluminescence (ECL) (Pierce, Rockford, IL, USA) system.

### 2.8. Immunofluorescence

The plate was washed with phosphate-buffered saline (PBS) once, and H9C2 cardiomyocytes were fixed with 4% paraformaldehyde for 30 minutes, and then, goat serum was added to sealing for 1 hour. The diluted primary antibody 8-hydroxydeoxyguanosine (8-OHdG, Abcam, Rabbit, 1 : 500) was added to incubate at 4°C overnight. The next day, the combined secondary antibody was incubated for 1 hour in the dark and stained with 4′,6-diamidino-2-phenylindole (DAPI) (Thermo Fisher Scientific, Shanghai, China) for nuclear staining. Finally, the film was sealed with a sealing liquid, and the image was observed under a fluorescence microscope (Olympus, Tokyo, Japan).

### 2.9. Enzyme-Linked Immunosorbent Assay (ELISA)

H9C2 cardiomyocytes were plated at 5,000/well in 6-well plates, and the cells were treated differently. The concentration of GPX1 and GPX3 in the cell supernatant was measured according to the instructions using an ELISA kit (Elabscience, Wuhan, China).

### 2.10. Reactive Oxygen Species (ROS) Level Detection

Flow cytometry was used to detect intracellular ROS levels. After different treatments of the three groups of cells, H9C2 cardiomyocytes were collected and washed with cold PBS. Total ROS levels were measured using flow cytometry (Becton Dickinson, Heidelberg, Germany) by intubation at 37°C for 20 min using DCFH-DA (10 M Kaiji, Nanjing, China).

### 2.11. Statistical Analysis

SPSS 21.0 statistical software (SPSS IBM, Armonk, NY, USA) was used to analyze the experimental data. Measurement data is expressed as *χ* ± *s*; *t*-test was used for comparisons between the two groups. Comparison between multiple groups was done using the one-way ANOVA test followed by the post hoc test (least significant difference). The LSD test or SNK test was used for pairwise comparison under the condition of homogeneity of variance. *p* < 0.05 indicated the significant difference. All experiments were repeated 3 times.

## 3. Results

### 3.1. Fasudil Retards Degradation of H9C2 Cardiomyocytes In Vitro

**Figure 1:**
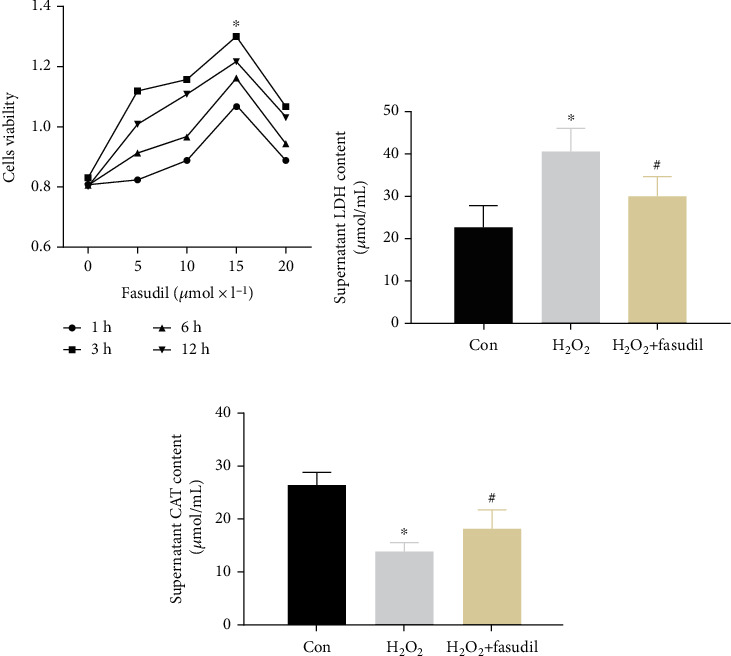
Fasudil retards degradation of H9C2 cardiomyocytes *in vitro*. (a) The optimal concentration and time of UTI were determined by the CCK-8 assay. (b) LDH kit detects the supernatant LDH content. (c) CAT kit detects the content of the supernatant CAT level (“∗” indicates statistical difference from the control group *p* < 0.05, and “#” indicates statistical difference from the ischemic hypoxia group *p* < 0.05).

The optimal concentration and optimal culture time point of fasudil-treated H9C2 cardiomyocytes were detected by CCK-8 (Figure 1(a)). We found that the highest cell viability was achieved after 3 hours of incubation with 20 *μ*mol∗L^−1^. LDH and CAT kits were used to detect cell supernatants. The results showed that LDH was significantly increased in the H_2_O_2_ group. CAT levels in the H_2_O_2_ group were significantly lower than in the Con group, while fasudil treatment significantly inhibited the reduction of CAT levels and inhibited the rise of LDH (Figures 1(b) and 1(c)).

### 3.2. Fasudil Inhibits Oxidative Stress Induced by H_2_O_2_ in H9C2 Cardiomyocytes

**Figure 2:**
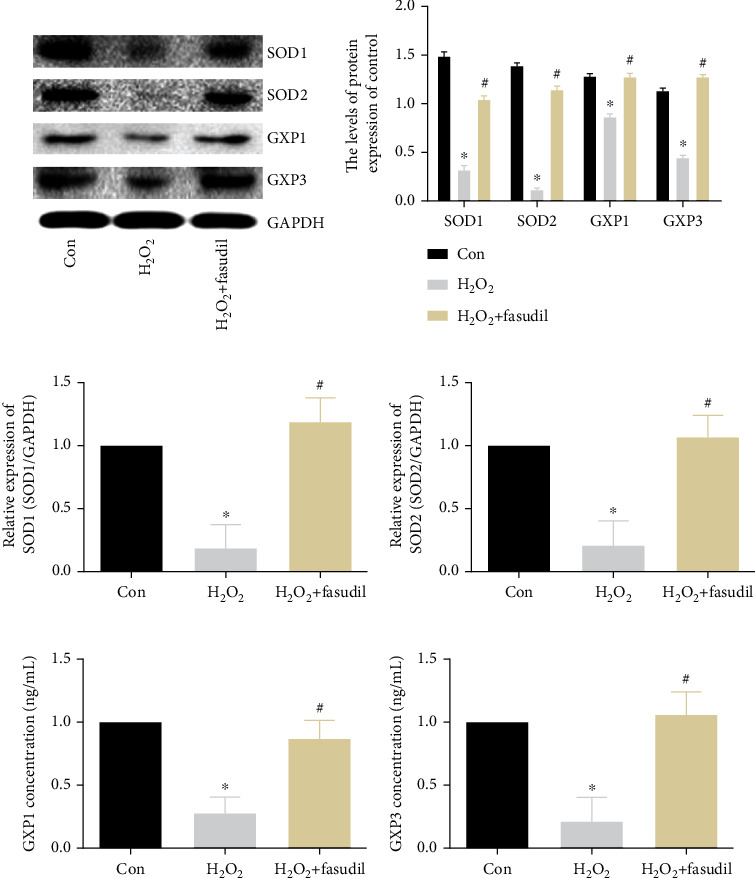
Fasudil inhibits oxidative stress induced by H_2_O_2_ in H9C2 cardiomyocytes. (a, b) Western blot was used to detect the expression of SOD1, SOD2, GPX1, and GPX3 in three groups. GAPDH was used as an internal control. (c, d) SOD1 and SOD2 mRNA expression was determined by real-time PCR. (e, f) ELISA detects the expression of GPX1 and GPX3 (“∗” indicates statistical difference from the control group *p* < 0.05, and “#” indicates statistical difference from the ischemic hypoxia group *p* < 0.05).

We detected antioxidant proteins by WB. Compared with the Con group, the expression of SOD1, SOD2, GPX1, and GPX3 in the H_2_O_2_ group was significantly decreased, while fasudil significantly promoted the expression of SOD1, SOD2, GPX1, and GPX3 (Figures 2(a) and 2(b)). The mRNA results were similar to the former: the SOD1 and SOD2 mRNA levels were decreased in the H_2_O_2_ group; compared with the Con group, the SOD1 and SOD2 mRNA levels in the H_2_O_2_+fasudil group were significantly increased (Figures 2(c) and 2(d)). ELISA results show that GPX1 and GPX3 were decreased in the H_2_O_2_ group, but the expression of GPX1 and GPX3 was obviously increased in the H_2_O_2_+fasudil group (Figures 2(e) and 2(f)).

### 3.3. Fasudil Inhibits H_2_O_2_-Induced Oxidative Damage in H9C2 Cardiomyocytes

**Figure 3:**
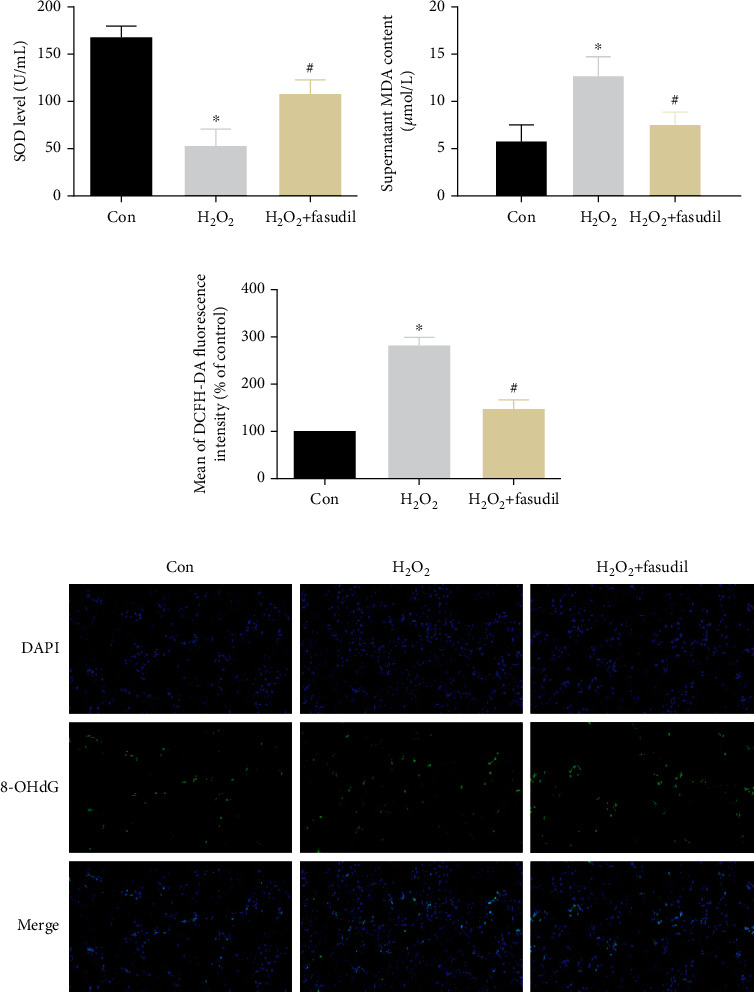
Fasudil inhibits H_2_O_2_-induced oxidative damage in H9C2 cardiomyocytes. (a) The SOD kit detects the level of cellular SOD. (b) The MDA kit detects the MDA content of the supernatant. (c) Flow cytometry was used to detect ROS levels. (d) Immunofluorescence was used to detect the expression of 8-OHdG in the three groups (“∗” indicates statistical difference from the control group *p* < 0.05, and “#” indicates statistical difference from the ischemic hypoxia group *p* < 0.05).

First, SOD and MDA levels were measured in the supernatant (Figures 3(a) and 3(b)). It was found that the SOD content was significantly decreased in the H_2_O_2_ group and significantly increased in the H_2_O_2_+fasudil group, while the MDA content was increased in the H_2_O_2_ group, and after fasudil treatment, the MDA content was decreased significantly. Flow cytometry results showed that the ROS levels were significantly increased in the H_2_O_2_ group, while ROS levels were significantly lower in the H_2_O_2_+fasudil group (Figure 3(c)). Immunofluorescence showed that the expression of 8-OHdG in the H_2_O_2_ group was significantly brighter than the Con group, while fasudil can effectively inhibit the expression of 8-OHdG in the H_2_O_2_+fasudil group (Figure 3(d)).

### 3.4. Fasudil Inhibits H_2_O_2_-Induced Apoptosis of H9C2 Cardiomyocytes

**Figure 4:**
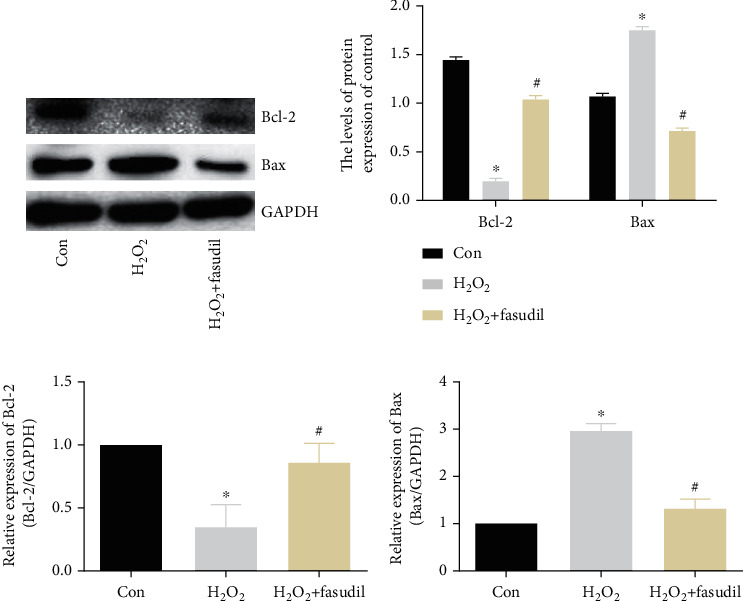
Fasudil inhibits H_2_O_2_-induced apoptosis of H9C2 cardiomyocytes. (a, b) Western blot was used to detect the expression of Bcl-2 and Bax in the three groups. GAPDH was used as an internal control. (c, d) Bcl-2 and Bax mRNA expression was determined by real-time PCR (“∗” indicates statistical difference from the control group *p* < 0.05, and “#” indicates statistical difference from the ischemic hypoxia group *p* < 0.05).

WB results showed that H_2_O_2_ treatment could induce apoptosis of H9C2 cardiomyocytes. The expression of Bax in the H_2_O_2_ group was significantly increased, while the expression of Bcl-2 was significantly inhibited. The treatment of H9C2 cardiomyocytes with fasudil can effectively inhibit the increase of Bax and promote the expression of Bcl-2 (Figures 4(a) and 4(b)). Similar levels were obtained for mRNA levels (Figures 4(c) and 4(d)).

### 3.5. Fasudil Inhibits H_2_O_2_-Induced Senescence of H9C2 Cardiomyocytes

**Figure 5:**
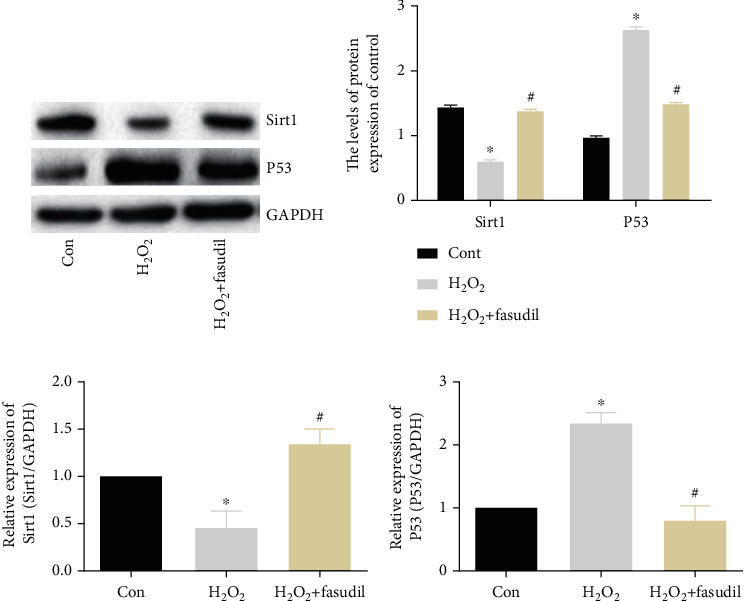
Fasudil inhibits H_2_O_2_-induced senescence of H9C2 cardiomyocytes. (a, b) Western blot was used to detect the expression of Sirt1 and P53 in the three groups. GAPDH was used as an internal control. (c, d) Sirt1 and P53 mRNA expression was determined by real-time PCR (“∗” indicates statistical difference from the control group *p* < 0.05, and “#” indicates statistical difference from the ischemic hypoxia group *p* < 0.05).

First, we detected senescence-associated proteins: Sirt1 and P53. Compared with the Con group, Sirt1 expression was significantly inhibited in the H_2_O_2_ group, and P53 expression was significantly promoted. In the H_2_O_2_+fasudil group, Sirt1 expression was significantly increased, and P53 expression was inhibited compared to the H_2_O_2_ group (Figures 5(a) and 5(b)). Second, Sirt1 mRNA was inhibited in the H_2_O_2_ group, while the P53 mRNA was significantly increased. However, in the H_2_O_2_+fasudil group, Sirt1 mRNA was higher than the H_2_O_2_ group, and P53 mRNA expression was inhibited (Figures 5(c) and 5(d)).

### 3.6. Fasudil Inhibits Rho/ROCK Pathway Activation

**Figure 6:**
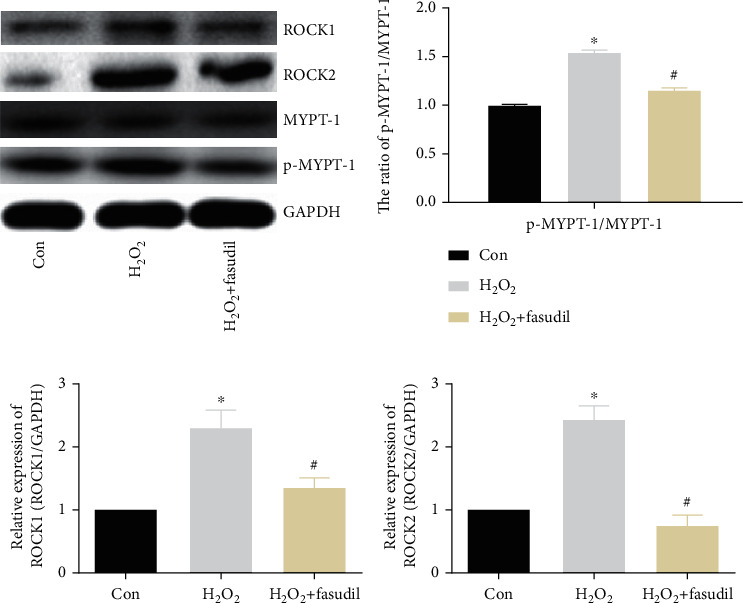
Fasudil inhibits Rho/ROCK pathway activation. (a) Western blot analysis of ROCK1, ROCK2, p-MYPT-1, and MYPT-1 expression in three groups. GAPDH was used as an internal control. (b) Protein gray value ratio of p-MYPT-1/MYPT-1. (c, d) ROCK1 and ROCK2 mRNA expression was determined by real-time PCR (“∗” indicates statistical difference from the control group *p* < 0.05, and “#” indicates statistical difference from the ischemic hypoxia group *p* < 0.05).

We found that ROCK1 and ROCK2 expression was significantly increased in the H_2_O_2_ group, and the expression of ROCK1 and ROCK2 was significantly inhibited in the H_2_O_2_+fasudil group. Next, we detected the downstream molecule MYPT-1 and p-MYPT-1, and the results showed that the expression ratio of p-MYPT-1/MYPT-1 was significantly increased in the H_2_O_2_ group, while fasudil can effectively inhibit the expression ratio of p-MYPT-1/MYPT-1 (Figure 6(a)). In addition, ROCK1 and ROCK2 mRNA was significantly elevated in the H_2_O_2_ group but was all inhibited by fasudil (Figures 6(c) and 6(d)).

## 4. Discussion

Previous studies have suggested that cell necrosis and apoptosis are the main ways of myocardial damage, but in recent years, extensive research confirmed that OS is another major way of myocardial cell damage [15]. Studies have found that a large number of ROS in the central and surrounding tissues of heart disease patients with heart disease indicate that OS is also one of the main causes of myocardial infarction [16]. Therefore, inhibition of cardiomyocyte OS response is essential for the prevention of cardiovascular disease.

To further investigate the mechanism of H_2_O_2_-induced OS in cardiomyocytes and to explore new interventions, we constructed a model by treating H9C2 cardiomyocytes with H_2_O_2_. Compared with the Con group, the antioxidant capacity of the H_2_O_2_ group was significantly reduced, and the apoptosis and senescence were significantly increased. As an inhibitor of the Rho/ROCK pathway, fasudil significantly increases the antioxidant capacity and effectively inhibits H_2_O_2_-induced apoptosis and senescence, as well as DNA damage. However, the molecular mechanism of fasudil in preventing H_2_O_2_-induced myocardial injury and its protective effects is still unclear and further research is needed. The results confirm that fasudil reduces H_2_O_2_-induced H9C2 cardiomyocyte OS by three aspects: (1) Fasudil improves cell clearance of ROS. (2) Fasudil can inhibit cell apoptosis, thereby reducing cell senescence. (3) Fasudil inhibits the Rho/ROCK signaling pathway and reduces H_2_O_2_-induced OS, thereby protecting H9C2 cardiomyocytes.

H_2_O_2_ is often used in cardiovascular disease models such as myocardial ischemia and myocardial ischemia and reperfusion [17]. Studies have shown that ROS can upregulate the expression of caspase family proteins by inhibiting Bcl-2 expression and promoting the transfer of Bax to mitochondria to induce the release of cytochrome C and initiate the mitochondrial apoptosis program [18]. The results of this experiment confirmed that Bax expression in the H_2_O_2_ group was significantly increased, but the Bax expression in the fasudil group was significantly inhibited, and the expression of antiapoptotic Bcl-2 was promoted.

Previous research confirmed that H_2_O_2_ produces OS damage to H9C2 cardiomyocytes. The ROS induced by H_2_O_2_ is cytotoxic, and ROS can cause destruction or even cleavage of DNA in the nucleus, eventually leading to apoptosis of a large number of H9C2 cardiomyocytes [19]. We confirmed by immunofluorescence that H_2_O_2_ treatment can significantly increase DNA damage in H9C2 cardiomyocytes, while the expression of 8-OHdG in the fasudil group was effectively inhibited, and slowing apoptosis.

A large number of articles reported that senescent was also one of the independent risk factors for cardiovascular disease. H_2_O_2_-induced oxidative damage can better simulate OS in the elderly, thereby promoting cell senescence [20]. This experiment verified the expression levels of senescence-associated molecule P53 and anti-senescence-related molecule Sirt1. Results show that Sirt1 expression was significantly decreased in the H_2_O_2_ group, while P53 expression was significantly increased. Fasudil treatment significantly inhibited the elevation of P53 and promoted the expression of Sirt1. The results showed that fasudil inhibited H_2_O_2_-induced H9C2 cardiomyocyte senescence, thereby alleviating H_2_O_2_ damage to H9C2 cardiomyocytes.

Rho kinase belongs to the serine/threonine protein kinase ACG family, which is an important effector downstream of RhoA of small GTP protein family [21]. Numerous researches have reported that the Rho/ROCK pathway is related in the regulation of inflammation and OS [22]. The results of this experiment show that H9C2 OS increased significantly after H_2_O_2_ treatment, which may be related to the activation of the Rho/ROCK pathway and the increase of downstream-related molecules. We confirmed that ROCK1 and ROCK2 expression in the H_2_O_2_ group and its downstream p-MYPT-1 was significantly prompted, while the ROCK kinase inhibitor fasudil was effective in inhibiting the increase of ROCK1, ROCK2, and p-MYPT-1 and inhibiting H_2_O_2_-induced H9C2 cardiomyocyte OS response.

## 5. Conclusions

Fasudil inhibits Rho/ROCK signaling and reduces OS, thereby inhibiting cell senescence and apoptosis. Therefore, fasudil is of great value in the intervention of hypoxic cardiomyopathy.

## Figures and Tables

**Table 1:** RT-PCR primers.

Gene name	Forward (5′ > 3′)	Reverse (5′ > 3′)
Bax	CAGTTGAAGTTGCCATCAGC	CAGTTGAAGTTACCATCAGC
Bcl-2	GACTGAGTACCTGAACCGGCATC	CTGAGCAGCGTCTTCAGAGACA
Sirt1	CCAGATCCTCAAGCCATG	TTGGATTCCTGCAACCTG
SOD1	GGTGAACCAGTTGTGTTGTC	CCGTCCTTTCCAGCAGTC
SOD2	CAGACCTGCCTTACGACTATGG	CTCGGTGGCGTTGAGATTGTT
P53	CAAAATGGTGAAGGTCGGTGTG	GATGTTAGTGGGGTCTCGCTC
ROCK1	AGATATGGCAAACAGGATT	CTTCACAAGATGAGGCAC
ROCK2	CTAGAGTGCCGTAGATGCCA	GGTTCTAGGGGATGATCGGG
GAPDH	ACAACTTTGGTATCGTGGAAGG	GCCATCACGCCACAGTTTC

qRT-PCR: quantitative reverse-transcription polymerase chain reaction.

## Data Availability

The datasets used and analyzed during the current study are available from the corresponding author on reasonable request.
